# Anorexia nervosa in adolescence from oral health perspective

**DOI:** 10.1192/j.eurpsy.2023.1113

**Published:** 2023-07-19

**Authors:** E. Paszynska, A. Hernik, M. Tyszkiewicz-Nwafor, M. Dmitrzak-Weglarz, M. Roszak, A. Slopien

**Affiliations:** 1 Department of Integrated Dentistry; 2 Department of Child and Adolescence Psychiatry; 3 Psychiatric Genetics Unit, Department of Psychiatry; 4 Department of Computer Sciences and Statistics, Poznan University of Medical Sciences, Poznan, Poland

## Abstract

**Introduction:**

Management of patients with anorexia nervosa (AN) desires psychiatric/medical care. In average AN disease onset they represent a younger generation than 18 y.o. In this age typically children and adolescents are under regular dental care. Whether young AN patients should be included to intensive oral supervision may be still questionable. In literature little information on changes in oral cavity caused by AN were reported.

**Objectives:**

Therefore, the aim of the study was to evaluate caries incidence, tooth wear, gingival inflammation, and oral hygiene level among adolescent AN inpatients, highlighting the aspect of oral health manifestations in case-control study.

**Methods:**

Based on clinically confirmed 130 AN restrictive subtype hospitalized female subjects (BMI <15 kg/m^2^, age 14.8±1.8), dental status has been examined regarding the occurrence of caries lesions using *Decay Missing Filling Teeth* (DMFT), erosive wear as *Basic Erosive Wear Examination* (BEWE), gingival condition as *Bleeding on Probing* (BOP) and plaque deposition as *Plaque Control Record* (PCR). The results were compared with age-matched 110 female controls (BMI 19.8±2.3 kg/m^2^, age 15.5±1.8, p=0.744) dentally caried in public University dental clinic (p<0.05) in the same time period.

**Results:**

AN patients compared with healthy adolescents were found to present higher incidence of oral-related complications according to dental status (DMFT 3.9±4.5 vs. 2.0±1.8, p=0.005), erosive tooth wear (BEWE 18.9% vs. 2.9%, p<0.001), less efficient in controlling plaque (PCR 43.8% vs. 13.7%, p<0.001) and gingival inflammation (20.0% vs. 3.9%, p<0.001). AN group, a significant correlation between BOP, BEWE, and duration of AN symptoms (p<0.05), similarly to the number of decayed teeth D, filled teeth F and PCR were detected (p<0.05).

**Conclusions:**

Although the obtained results did not reveal any severe oral complications, AN diagnosis in adolescence may influence to numerous oral-related symptoms from dental caries, the onset of erosive tooth wear, failure in dental hygiene to be continuated as gingival inflammation. After AN diagnosis a regular preventive intervention should be performed during dental recall sessions. There is a need for professional oral hygiene/diet instructions combined with regular oral check up visits to avoid oral complications and disease progress. For clinical revelance an active collaboration between psychiatric and dental specialists is needed.

**Disclosure of Interest:**

None Declared

